# Supplementary material for: Reverse Chromatin Immunoprecipitation (R-ChIP) enables investigation of the upstream regulators of plant genes
Source: Commun Biol. 2020 Dec 14;3:770. doi: 10.1038/s42003-020-01500-4 (PMC7736860; doi:10.1038/s42003-020-01500-4)
Supplement: Supplementary file 2 — Supplementary Information [file 42003_2020_1500_MOESM2_ESM.pdf]

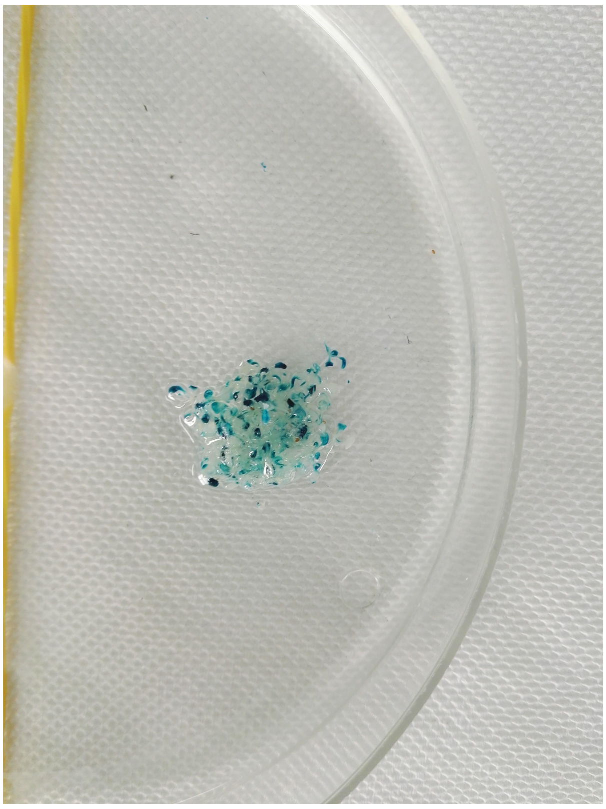

Supplementary Fig. 1: The full and uncropped image of Fig. 5a.
Investigation of the efficiency of transient transformation by GUS staining. The *Arabidopsis* plants were transiently transformed with pCAMBIA1301. After transformation for 72 h, GUS staining was performed.

The promoter of *AtCAT3* (AT1g20620)

gattgtcttctaatgattgccactttctgagaatagtatgttactgttgattgttgtacctagctctagttgtaaataataatatcaaa
-1281bp
ctaagtaacaagagcaacttgttgacactaaagggaccccaagttaccctcaatcaaaaattgtggatcagttcatcatcgaa

gtaacgtgttaattatcgtcatatgctccacaacatcgactccccaccatacccactcaataatgtcaattcagctctctcttttg

tatttttataaagaatgtatttaaattttcaacccataatttatagattcatgatcatctattaataattcattaaatacttttctgcatatt

gatttatttaaaagccgtacgcaagctcaaatcggataagactaaaataatatcacccagttaagttaactaacttattttattttg
bio-ATCAT3p-12
aatagtagtattaattacttttgttcaactactccaatactgtttatatcctttcgacggtattagttattttcatgtttcacgcatttttgt
bio-ATCAT3p-4
aattgcaaatacagacacctttgttgtaacacatgttgattaattttccgaaactatatacttttgaagacaaaaatttaaaacaaa

gaaacctcagatctacagttccacctcgtgtctgacacgagtttctatgcgataagatcgaacatgtatatcagcagactttact

atagatgaactcatattagtaataagatccaaatacggaaataaacaacttggaaagtgaatggaagttttaagagaaaaatc
bio-ATCAT3p-11
atttctagaattattttttagatcatttcttttttggagcatccattagagttttgtttttagactttatgatttatttttcttttgttatcagc
bio-ATCAT3p-3
acaataattgattttcaaaagatattgtatcttttttttatctcttgagaagcacagcacaacaacgagcaatgctgacttgtcgg
bio-ATCAT3p-10
ggtcagcgattattattagtcaccgaacgaatttttcttgggttggagccaaaacctagcaaaaaaatatctaagtgggggca
bio-ATCAT3p-2 **bio-ATCAT3p-9**
ctacattattgttttaacaccaaggaaaagtaaacaacaatggcccatttttactgtatcgagaaattgatctcgaaaggacaa
bio-ATCAT3p-8
atgtacccttccacttcgggaatccattattgtgcataacacacgctgtttcactatatatactttcacataggatctcaacactct
bio-ATCAT3p-1 **bio-ATCAT3p-7**
cactaaaccaaaatcctcaaaagcctatttggggggatcatcaaccttctatcatcaccATGGATCCTTACAAG
bio-ATCAT3p-6 **bio-ATCAT3p-5** **0 bp**

Supplementary Fig. 2: The location and length information of biotin probes used for hybridization.

The figure shows the sequence of the promoter of *AtCAT3*, which is 1281 bp upstream of translation start site. The arrow indicates the location, sequence, and the direction information of 12 probes that were labeled with biotin and used for hybridization.

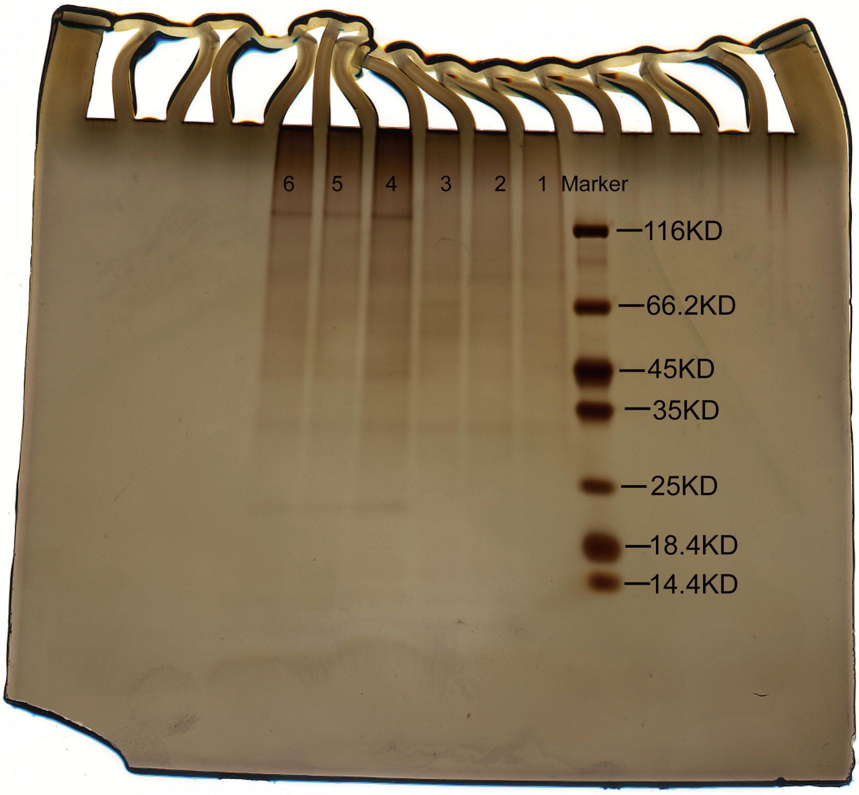

Supplementary Fig. 3: The full and uncropped gel image of Fig. 6b.

SDS-PAGE analysis of the proteins captured using R-ChIP. Lanes 1–3: the proteins isolated from wild-type plants from three biological replicates, respectively. Lanes 4–6: the proteins isolated from transient transformed plants from three biological replicates, respectively. The proteins were stained with silver nitrate.

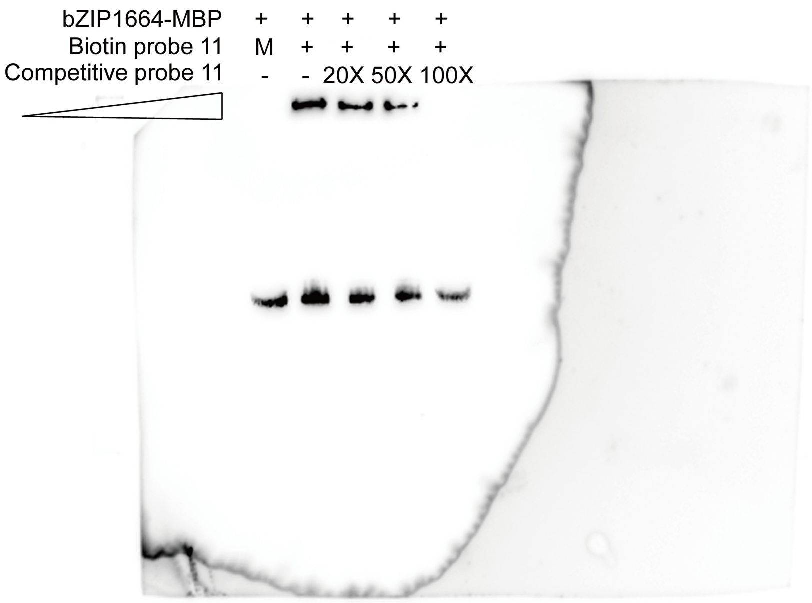

Supplementary Fig. 4: The full and uncropped blot image of Fig. 7g.

The binding of bZIP1664 protein to the promoters of *AtCAT3* as assessed using EMSA. M: mutant probe was the promoter of *AtActin3*.

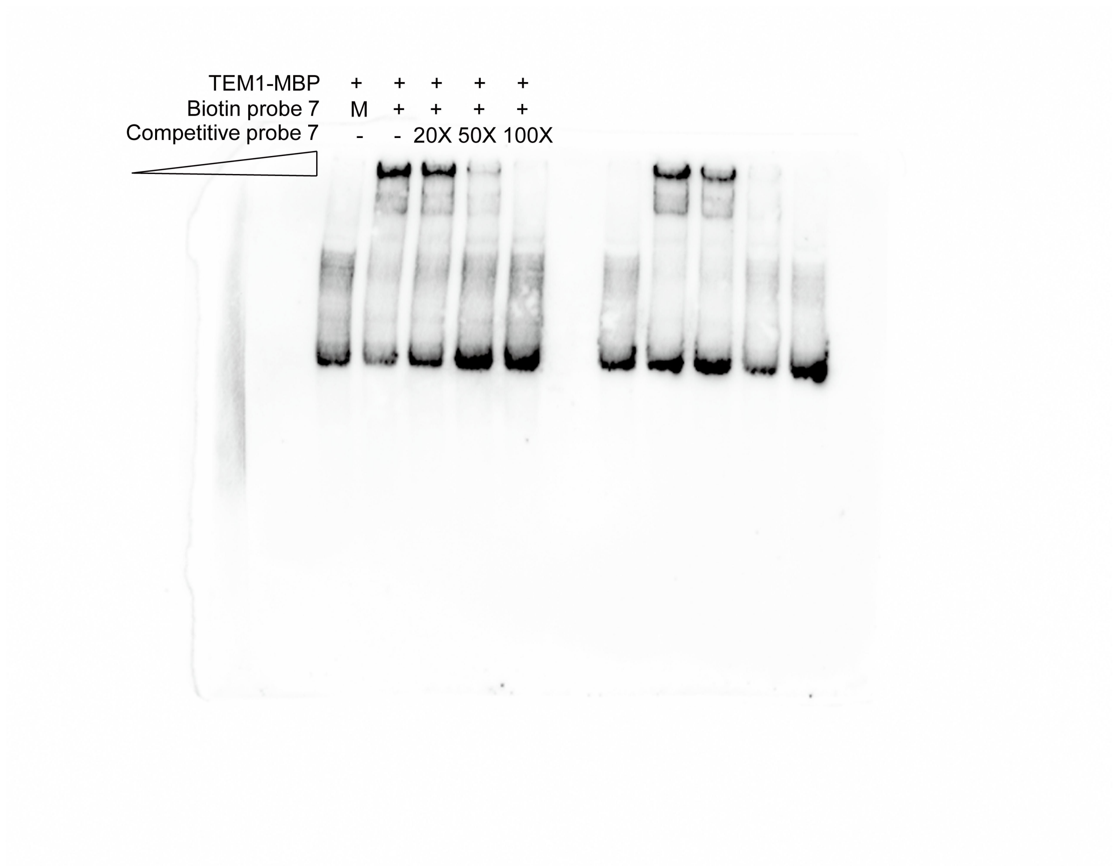

Supplementary Fig. 5: The full and uncropped blot image of Fig. 7h.

The binding of TEM1 protein to the promoters of *AtCAT3* as assessed using EMSA. M: mutant probe was the promoter of *AtActin3*.

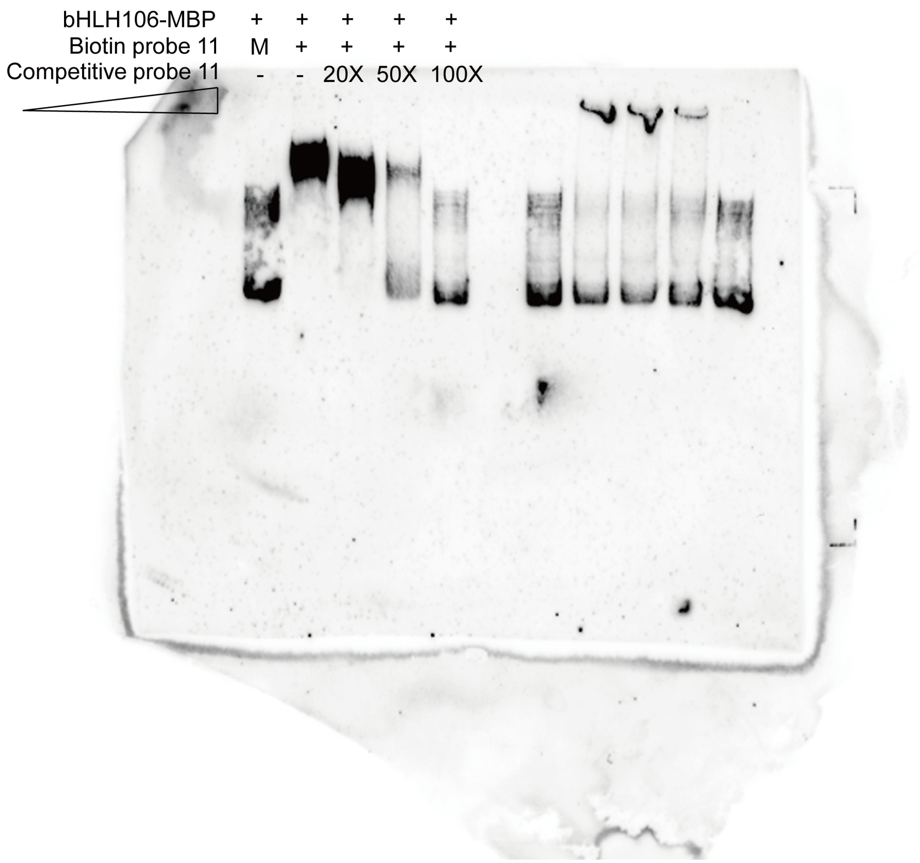

Supplementary Fig. 6: The full and uncropped blot image of Fig. 7i.

The binding of bHLH106 protein to the promoters of *AtCAT3* as assessed using EMSA. M: mutant probe was the promoter of *AtActin3*.

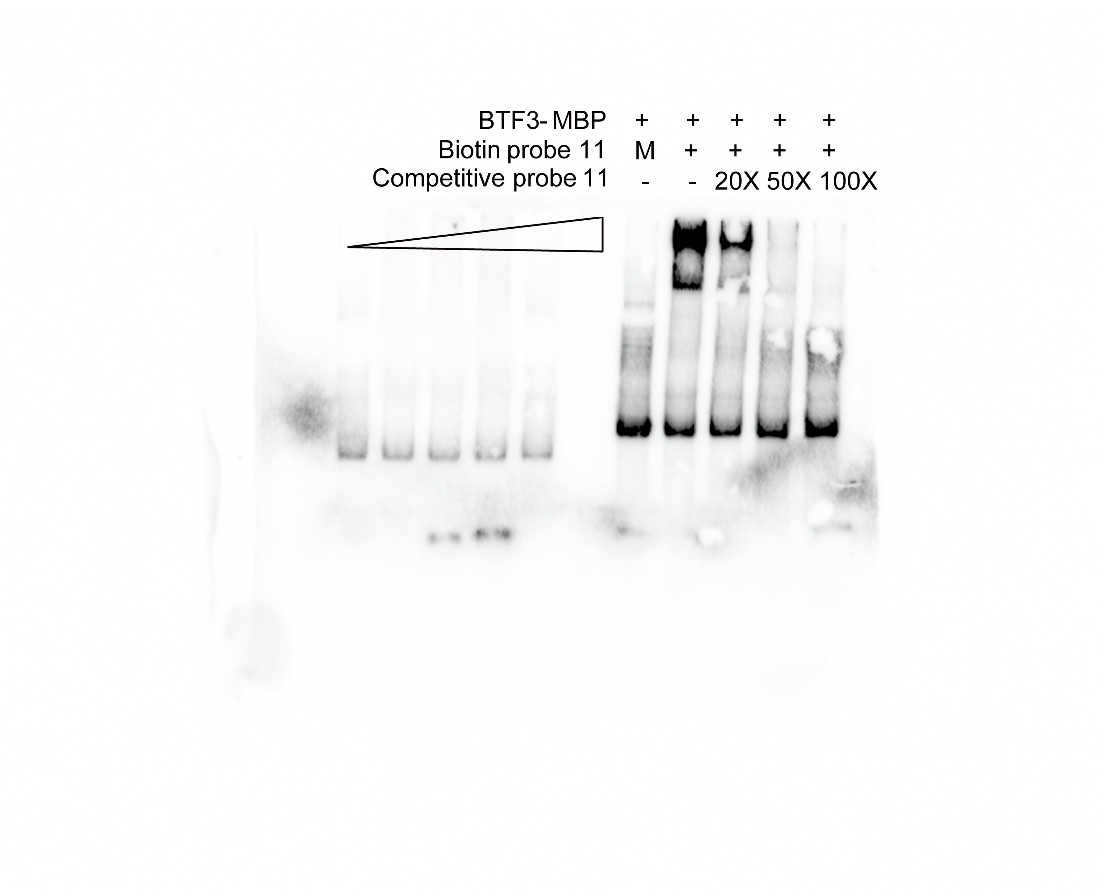

Supplementary Fig. 7: The full and uncropped blot image of Fig. 7j.

The binding of BTF3 protein to the promoters of *AtCAT3* as assessed using EMSA. M: mutant probe was the promoter of *AtActin3*.

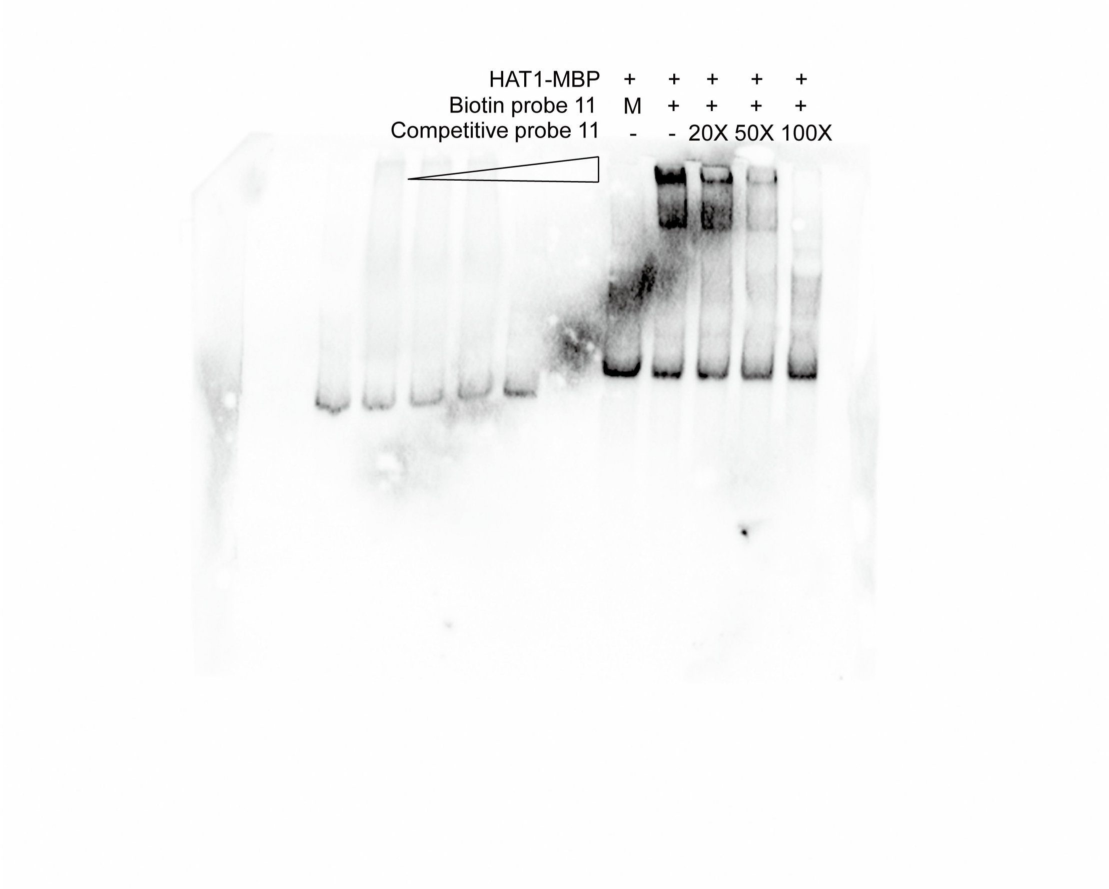

Supplementary Fig. 8: The full and uncropped blot image of Fig. 7k.

The binding of HAT1 protein to the promoters of *AtCAT3* as assessed using EMSA. M: mutant probe was the promoter of *AtActin3*.
