# Supplementary material for: Reverse Chromatin Immunoprecipitation (R-ChIP) enables investigation of the upstream regulators of plant genes
Source: Commun Biol. 2020 Dec 14;3:770. doi: 10.1038/s42003-020-01500-4 (PMC7736860; doi:10.1038/s42003-020-01500-4)
Supplement: Supplementary file 3 — Description of Additional Supplementary Files [file 42003_2020_1500_MOESM3_ESM.pdf]

Description of Additional Supplementary Files

File Name: Supplementary Data 1
Description: Mass spectrometry results of wild-type and transiently transformed samples (3 biological replicates).

File Name: Supplementary Data 2
Description: List of transcription regulators obtained from mass spectrometry results.

File Name: Supplementary Data 3
Description: The enrichment folds of all studied TFs in different regions of AtCAT3 promoter.

File Name: Supplementary Data 4
Description: The length and location information of sites studied in ChIP and EMSA.

File Name: Supplementary Data 5
Description: All source data underlying the graphs and charts in the main figures.

File Name: Supplementary Data 6
Description: The primers used for vector construction.

File Name: Supplementary Data 7
Description: The sequences of probes and primers used for hybridization and EMSA.

File Name: Supplementary Data 8
Description: The primers used for qPCR.
